# Impact of electronic medical record on physician practice in office settings: a systematic review

**DOI:** 10.1186/1472-6947-12-10

**Published:** 2012-02-24

**Authors:** Francis Lau, Morgan Price, Jeanette Boyd, Colin Partridge, Heidi Bell, Rebecca Raworth

**Affiliations:** 1 School of Health Information Science, University of Victoria, P.O. Box 3050 STN CSC, Victoria V8W3P5, Canada; 2 Faculty of Medicine, University of British Columbia, 5950 University Blvd, Vancouver V6T1Z3, Canada; 3 Admirals Medical Clinic, 275 Island Hwy, Victoria V9B1G4, Canada; 4 Kootenay Boundary and Creston Community of Practice, 518 Lake Street, Nelson V1L4C6, Canada; 5 University of Victoria Libraries, University of Victoria, P.O. Box 1800 STN CSC, Victoria V8W3H5, Canada

## Abstract

**Background:**

Increased investments are being made for electronic medical records (EMRs) in Canada. There is a need to learn from earlier EMR studies on their impact on physician practice in office settings. To address this need, we conducted a systematic review to examine the impact of EMRs in the physician office, factors that influenced their success, and the lessons learned.

**Results:**

For this review we included publications cited in Medline and CINAHL between 2000 and 2009 on physician office EMRs. Studies were included if they evaluated the impact of EMR on physician practice in office settings. The Clinical Adoption Framework provided a conceptual scheme to make sense of the findings and allow for future comparison/alignment to other Canadian eHealth initiatives.

In the final selection, we included 27 controlled and 16 descriptive studies. We examined six areas: prescribing support, disease management, clinical documentation, work practice, preventive care, and patient-physician interaction. Overall, 22/43 studies (51.2%) and 50/109 individual measures (45.9%) showed positive impacts, 18.6% studies and 18.3% measures had negative impacts, while the remaining had no effect. Forty-eight distinct factors were identified that influenced EMR success. Several lessons learned were repeated across studies: (a) having robust EMR features that support clinical use; (b) redesigning EMR-supported work practices for optimal fit; (c) demonstrating value for money; (d) having realistic expectations on implementation; and (e) engaging patients in the process.

**Conclusions:**

Currently there is limited positive EMR impact in the physician office. To improve EMR success one needs to draw on the lessons from previous studies such as those in this review.

## Background

### The need

Increased investments are being made for electronic medical record (EMR) systems to improve physician practice in office settings in Canada. Physician office-based EMR funding support programs are in place in several Canadian provinces [1] and recently there is a pan-Canadian "EMRs and Integration" investment program from Canada Health Infoway [2]. The potential value for EMRs is widely acknowledged, including improved office productivity, care coordination, and patient safety [3]. Yet significant challenges remain in adopting office-based EMRs and reaping the benefits. For instance, two Canadian EMR studies have shown that physicians underestimate the need for substantive time commitment, EMR-savvy champions, and ongoing technical/funding support [4,5]. Given the current rates of deployment of EMRs in Canada and what is at stake, there is an urgent need to learn from previous EMR studies to determine what had made them successful.

There are systematic reviews on the use of information technology (IT) including EMRs in primary care and general practice settings. These reviews covered topics in diabetes management [6], patient record quality [7,8], decision support tools [9], electronic communication [10], and provider performance and patient outcomes [11]. While there is some evidence of improved quality in such areas as preventive care and guideline adherence, many challenges have been reported [9-11]. These include variable consistency and accuracy of patient record content [7,8], lack of time and funding to cope with change, and the need for adequate training and support [6]. While informative, the shortcomings of these reviews are that they were mostly in specific topic areas based on controlled trials published before 2005 (except for [6,9] which were published in 2008). Some of these reviews had a mix of inpatient and outpatient settings, and included large Health Maintenance Organizations with EMRs that are integrated across multiple hospitals and ambulatory care clinics. In contrast, most Canadian physician offices tend to be privately owned solo/group practices or interdisciplinary community-based clinics with standalone EMR systems from small/medium size vendors that are not well integrated with the other health information systems. Different approaches have also been applied to examine the impact of EMRs, including field observation studies [12], workflow analysis [13] and surveys [14]. Thus, there is a need to conduct an EMR review for office settings similar to those in Canada and be more inclusive of different evaluation approaches, covering multiple topic areas [15,16].

This paper describes a systematic review we conducted on EMR-supported physician practice in the office setting. Our questions were: (a) What is the impact of EMRs on physician office? (b) Is there a difference in impact by country, time period and study design? (c) What factors may have led to such impact in the office? (d) What overarching lessons can be drawn to improve EMR success in office-based physician practice? In this review, the Clinical Adoption Framework by Lau et al. [17] was used to make sense of EMR impact and success.

### Conceptual model

**Figure 1:**
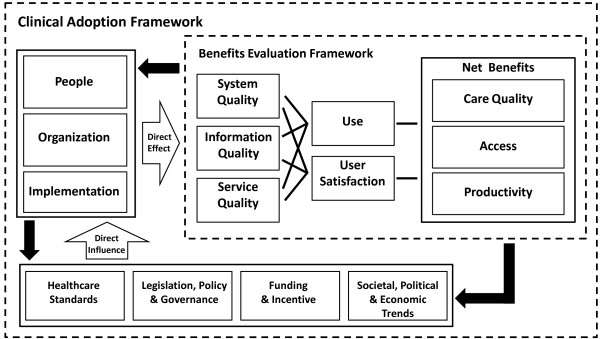
**Clinical Adoption Framework with the micro, meso and macro dimensions that affect HIS success **[17].

The Clinical Adoption (CA) Framework provides a conceptual model to describe the factors that influence health information systems (HIS) success (Figure 1). It extends the Infoway Benefits Evaluation (BE) Framework published in 2006 [18] (adapted from the DeLone and McLean information system success model [19,20]). The CA Framework is comprised of micro, meso and macro-level dimensions. At the micro-level, the CA Framework describes HIS success in terms of HIS quality, use and net benefits. HIS quality includes information, system and service quality; use includes HIS usage and satisfaction; net benefits include care quality, access, and productivity. At the meso-level HIS success is influenced by people, organization and implementation factors. At the macro-level, HIS success is influenced by healthcare standards, legislation/policy/governance, funding/incentive and socio-political and economic trends. The CA Framework was developed with a broad range of HIS in mind, including EMRs.

In this review, we examined EMR impact and success in office settings thru the lens of the CA Framework. We defined impact as EMR adoption and effect on physician practice, based on evaluation measures used in the studies. For factors that led to such impact we defined them as the observations and/or reasons cited that could explain the adoption and effect. For EMR success we defined it as the improvement that EMRs can make in the physician office. This was similar to reviews that assessed the effects of HIS in other health settings [21,22].

## Results

### Synopsis of selected studies

**Figure 2:**
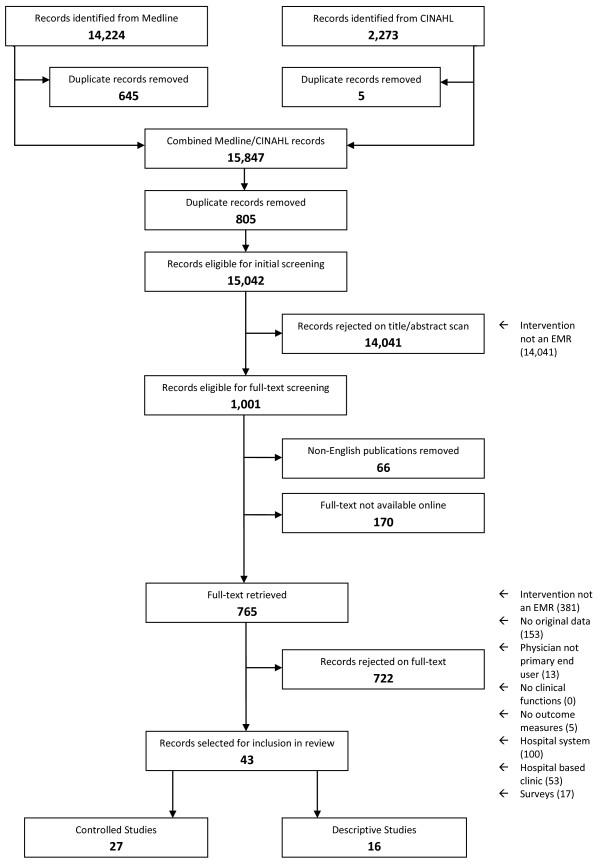
**A flow chart of the study selection process and results**.

Our initial search returned 15,042 unique citations. Screening of titles and abstracts left 1,001 articles that required review. While retrieving the full-text for these studies, we removed 66 non-English publications and 170 citations that were not available online (the review team decided not to pursue them due to time constraint). Of the 765 full-text articles screened, 43 studies were selected for this review (27 controlled, 16 descriptive) [12,13,23-63]. See Figure 2.

A synopsis of the 43 studies is shown in Additional file 1. Twenty-seven of the 43 studies (62.8%) were published between 2005 and 2009. Studies from the United States (11 or 25.6%) and United Kingdom (10 or 23.3%) made up half the publications. The remaining studies were from The Netherlands (5 or 11.6%), Canada (4 or 9.3%), Australia (4 or 9.3%), Norway (2 or 4.6%), New Zealand (2 or 4.6%), and other countries. Fourteen of 43 studies (32.6%) were focused on work practice, 9 (20.9%) on prescribing support, 7 (16.3%) on disease management, 6 (13.9%) on clinical documentation, 4 (7.0%) on preventive care and 4 (9.3%) on patient-physician interaction. For study design, 11 (25.6%) were randomized controlled trial, 7 (16.3%) quasi-experimental, 9 (20.9%) observational and 16 (37.2%) qualitative studies. The observational studies included 6 cross-sectional cohort, 1 time-motion, 1 prospective audit and 1 secondary analysis studies. The qualitative studies included 6 multi-method designs, 6 case series, 3 videotape analyses and 1 interview study.

### EMR impacts by study and measure

The 43 studies are summarized by topic area, impacts, influencing factors and our mapping to the CA Framework in Additional file 2. The differences in impact by country, time period and design are reported in Additional file 3. When compared between countries with high versus low adoption rates there was no significant difference found in the ratios of positive studies. There was no difference found between the two time periods of 2000-04 and 2005-09. Odds ratio tests showed more positive results (but not significant) from controlled-experimental than controlled-observational studies, and no difference between controlled and descriptive studies.

Table 1 summarizes the impacts by study for the six topic areas. For controlled studies, the number of positive counts ranged from highest in work practice (4/5 studies or 80.0%) to lowest in clinical documentation (1/5 studies or 20.0%). For descriptive studies, the positive count was lower in work practice (5/9 studies or 55.6%), while the remaining areas had too few study counts for meaningful comparison. When combined, 22/43 studies (51.2%) had positive impacts, 13/43 studies (30.2%) had neutral impacts and 8/43 studies (18.6%) had negative impacts. The areas with > 50% positive counts were preventive care (2/3 studies or 66.7%), work practice (9/14 studies or 64.3%) and disease management (4/7 studies or 57.1%). The area with the most negative counts was clinical documentation (3/6 studies or 50.0%).

**Table 1:** Number of positive, neutral and negative impacts by study for the six areas

Topic Areas	Positive (%)	Neutral (%)	Negative (%)	Total
**Controlled**				
Prescribing Support	3 (37.5)	4 (50.0)	1 (12.5)	8
Disease Management	4 (57.1)	2 (28.6)	1 (100.0)	7
Clinical Documentation	1 (20.0)	2 (40.0)	2 (40.0)	5
Work Practice	4 (80.0)	1 (20.0)	0 (0.0)	5
Preventive Care	1 (33.3)	1 (50.0)	0 (0.0)	2
Patient-Physician Interaction	0 (0.0)	0 (0.0)	0 (0.0)	0
Sub-total	13 (48.1)	10 (37.0)	4 (14.8)	27
**Descriptive**				
Prescribing Support	1 (100.0)	0 (0.0)	0 (0.0)	1
Disease Management	0 (0.0)	0 (0.0)	0 (0.0)	0
Clinical Documentation	0 (0.0)	0 (0.0)	1 (100.0)	1
Work Practice	5 (55.6)	1 (11.1)	3 (33.3)	9
Preventive Care	1 (100.0)	0 (0.0)	0 (0.0)	1
Patient-Physician Interaction	2 (50.0)	2 (50.0)	0 (0.0)	4
Sub-total	9 (56.3)	3 (18.8)	4 (25.0)	16
**Combined**				
Prescribing Support	4 (44.4)	4 (44.4)	1 (11.1)	9
Disease Management	4 (57.1)	2 (28.6)	1 (14.3)	7
Clinical Documentation	1 (16.7)	2 (33.3)	3 (50.0)	6
Work Practice	9 (64.3)	2 (14.3)	3 (21.4)	14
Preventive Care	2 (66.7)	1 (33.3)	0 (0.0)	3
Patient-Physician Interaction	2 (50.0)	2 (50.0)	0 (0.0)	4
Total*	22 (51.2)	13 (30.2)	8 (18.6)	43

*Odds ratios between controlled and descriptive studies are not significant

The impacts are grouped according to the micro-level dimensions of the CA Framework in Table 2. Odds ratio testing showed only minor differences between the controlled and descriptive studies (i.e., neutral impact, OR = 2.5 CI 1.1-5.9). For controlled studies only productivity had > 50% positive count with 10/16 measures (62.5%) positive. For descriptive studies the three measures that had > 50% positive counts were care quality (4/4 measures; 100.0%), information quality (4/6 measures; 66.7%) and productivity (11/17 measures; 64.7%) that were positive. When combined, 50/109 measures (45.9%) showed positive impacts, 39/109 measures (35.8%) showed neutral impacts and 20/109 measures (18.3%) showed negative impacts. Overall, the only measure that had > 50% positive count was productivity where 21/33 measures (63.6%) were positive.

**Table 2:** Number of positive, neutral and negative impacts by measure in the CA Framework

Topic Areas Controlled	Positive (%)	Neutral (%)	Negative (%)	Total
Info System Quality				
System	1 (33.3)	0 (0.0)	2 (66.7)	3
Information	4 (33.3)	6 (50.0)	2 (16.7)	12
Service	0 (0.0)	0 (0.0)	0 (0.0)	0
Use/Satisfaction				
User Satisfaction	0 (0.0)	0 (0.0)	0 (0.0)	0
Net Benefits				
Care Quality	12 (37.5)	16 (50.0)	4 (12.5)	32
Access				0
Productivity	10 (62.5)	6 (37.5)	0 (0.0)	16
Subtotal	27 (42.9)	28 (44.4)	8 (12.7)	63
**Descriptive**				
Info System Quality				
System	2 (33.3)	0 (0.0)	4 (66.7)	6
Information	4 (66.7)	1 (16.7)	1 (16.7)	6
Service	0 (0.0)	0 (0.0)	2 (100.0)	2
Use/Satisfaction				
User Satisfaction	2 (18.2)	9 (81.8)	0 (0.0)	11
Net Benefits				
Care Quality	4 (100.0)	0 (0.0)	0 (0.0)	4
Access	0 (0.0)	0 (0.0)	0 (0.0)	0
Productivity	11 (64.7)	1 (5.9)	5 (29.3)	17
Subtotal	23 (50.0)	11 (23.9)	12 (26.1)	46
**Combined**				
Info System Quality				
System	3 (33.3)	0 (0.0)	6 (66.7)	9
Information	8 (44.4)	7 (38.9)	3 (16.7)	18
Service	0 (0.0)	0 (0.0)	2 (100.0)	2
Use/Satisfaction				
User Satisfaction	2 (18.2)	9 (81.8)	0 (0.0)	11
Net Benefits				
Care Quality	16 (44.4)	16 (44.4)	4 (11.1)	36
Access	0 (0.0)	0 (0.0)	0 (0.0)	0
Productivity	21 (63.6)	7 (21.2)	5 (15.2)	33
**Total**	**50 (45.9)**	39 (35.8)*	**20 (18.3)**	**109**

*Significant odds ratio (OR) = 2.5, CI 1.10-5.90

### Factors influencing EMR adoption and effect

**Figure 3:**
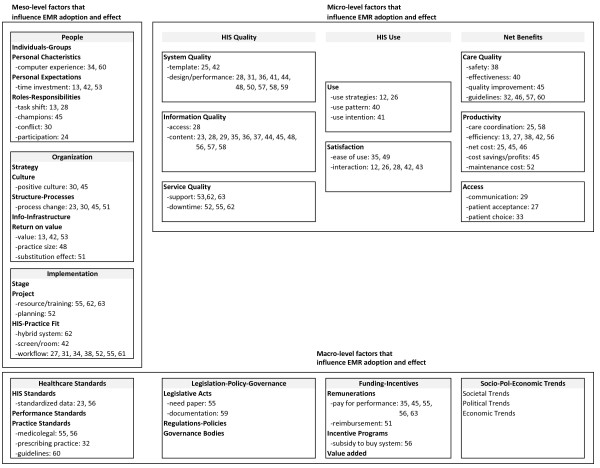
**Micro, meso and macro-level factors that influenced EMR success**.

A total of 100 factors that influenced EMR adoption and its effect were identified from the 43 studies (see Additional file 2). After merging those that were similar we ended up with 48 distinct factors. These factors were mapped to the categories of the CA Framework [refer to [17,18]]: 23 of them were micro-level, 16 meso, and 9 macro (see Figure 3). The types of influence are elaborated below.

At the *micro-level*, system quality factors included the availability of templates [25,42], interface design [31,36,41,44,48,50,55,57,59] and technical performance (e.g. speed and reliability) [44,55,58]. Information quality factors included the organization, accuracy, completeness and accessibility of the patient record [23,28,29,35-37,44,45,47-56]. Service quality factors included training and technical support [53,62,63], system backup and unexpected downtime [52,55]. EMR usage factors included its intent (e.g. quality improvement versus record keeping) [41], actual strategies for optimal/appropriate use [12-26], ease of use [35,49], and usage patterns that emerged over time [40]. Interaction related factors covered patient-physician encounters such as the type of consult (e.g. psychological) [12,26,42,43], consult room layout [42] and patients' ability to schedule appointments [27,33]. For net benefits, care quality factors covered patient safety [38], care effectiveness [40], quality improvement [45] and guideline compliance [32,57,60,61]. Productivity factors covered care efficiency [13,27,38,42,56], coordination [24,58] and net cost including billing, staffing and maintenance costs [25,45,46,52].

At the *meso-level*, people factors included personal characteristics and expectations such as prior EMR experience of the users [34,60], and their personal time investment in exchange for the benefits expected from the system [13,28,42]. Roles/responsibilities covered the need for champions and staff participation [24,45], and shift in tasks (e.g. documentation by staff vs. physicians) [13,28] that could lead to role ambiguity and conflict [30]. Organization factors included structure/processes and culture that supported EMR adoption/use [23,30,45,51], EMR-practice fit (e.g. hybrid EMR/paper systems) [50], and EMR-supported office and workflow design [30,45,51,53,56,59,63] such as the placement of computer screens in consult rooms [42]. Return-on-value focused on demonstrated value at the practice level such as substitution effect from guideline driven test orders and prescribing [51], and tangible cost-efficiency gain with larger practice size and patient volume [48]. Implementation factors included the extent that the introduction of an EMR into the practice was planned and carried out as a priority project with dedicated time and resources [52,55,62,63]. The service support provided during implementation was critical [48,53,62,63], since they affected the disruptions that physicians and office staff had to overcome while learning to use the EMR and redesign their work routines.

At the *macro-level*, factors under healthcare standards included standardized data content [23,56], established practice guidelines [32,60], and legal documentation requirements [55,56,59] that affected EMR design/performance and user behaviours. They also covered practice standards for clinical guidelines [60], professional scope of practice [32] and medico-legal requirements [55,56] that governed EMR use. Factors under funding/incentives included remuneration schemes such as pay for performance and fee-for-service that encouraged EMR use [35,45,55,56,63] and incentive programs in the form of subsidies to purchase/adopt EMR systems [56].

### Summary of key findings

Overall, this review found 22/43 EMR studies (51.2%) and 50/109 measures (45.9%) had shown positive impact across the six topic areas examined. When grouped by area, there were modest improvements in preventive care (66.7%), work practice (64.3%) and disease management (57.1%). Clinical documentation showed the least improvement with EMR use (16.7%). Within the dimensions of the CA Framework, EMRs had shown a modest improvement in productivity (63.6%), whereas user satisfaction had the least improvement (18.2%). About one-third of the studies and measures were not able to show an impact. Less than one-fifth of the studies and measures had a negative impact. No significant differences were found based on adoption rates by country, by time period and by study design.

Through this review we found that EMR impact was influenced by many factors. In particular, we were able to extend the CA Framework to EMRs in physician office settings by identifying specific micro, meso and macro level factors that influence EMR adoption and its effect. For instance, at the micro-level, the EMR's technical design, performance and support affected its usage and user satisfaction in the office. At the meso-level, the implementation process and resulting workflow impacted the office's ability to improve productivity and coordination. At the macro-level, incentives such as pay-for-performance were seen as an important driver for EMR adoption since they increased the return on investment made.

## Discussion

### Making sense of EMR success

Our review findings suggest there is a 51% chance that an EMR can improve office practice, while in 30% of the time there may not be any effect, but only 19% may lead to negative consequences. Surprisingly, no significant differences in impact were found from studies across countries, time periods and study designs. When compared with the earlier reviews for primary care and general practice settings, our findings showed less improvement in the areas of disease management [6], patient record quality [7,8] and decision support tools [9], but similar improvement in preventive care [11]. When compared with the more general HIS review by Lau et al. [15], which showed reported positive impacts in 62.7% of studies and 54.4% of measures, our counts looking at EMRs were lower at 51.2% and 45.9%, respectively. There was similar improvement in preventive care impacts (66.7% vs. 72.1%) [15], while our review showed greater improvement in work practice (64.3%).

A further comparison was made with an earlier review by Eslami et al. [64] on inpatient computerized provider order entry (CPOE) systems identified in the Lau review [15]. The inpatient CPOEs had greater positive impacts in guideline adherence, organizational efficiency and user satisfaction than those reported in our EMR review. These findings suggest that there are differences for physicians in office settings that need to be considered; perhaps they face greater challenges with less advanced EMR features and fewer resources available for improvement. While these comparisons place our review results into larger HIS contexts, caution is needed when interpreting these findings since the range of studies, methods, systems and settings examined were highly variable across these reviews.

### Extending the CA framework to EMR in office settings

This review had extended the CA Framework by identifying specific factors that influenced EMR success in the office setting. These factors are also consistent with those reported in the earlier reviews. Examples are the need for adequate time, funding and training [6], accurate clinical documentation [7,8] and computer-supported interactions among physicians and patients [10,11]. Other factors are the importance of having computer-prompted alerts/reminders and involving users in system design [21]. When compared with the success factors reported in van der Meijden's review [65] of inpatient clinical information systems, we were able to map many of their factors to those listed under the micro-level dimensions of the CA Framework in our EMR review. In addition, we were able to map all of their contingent factors to those in the meso and macro levels of the CA Framework in our review. These findings suggest that, the adoption and ongoing use of office-based EMRs face many of the same issues as other HIS deployment efforts, although the ways of addressing these issues may be different. In this regard, the CA Framework should be expanded by incorporating post-adoption usage behaviours by physicians and their effects over time to reflect the different maturity stages of the EMRs involved [66,67].

### Lessons to guide future efforts

Based on this review we argue there is much room for improvement. Specifically, we can achieve EMR success by drawing on the lessons from earlier studies examined in this review. The key lessons are: (1) *Having robust EMR features that support ongoing clinical use *- by paying close attention to interface design (e.g. templates, decision support) and technical performance (e.g. reliability and speed) issues to ensure the efficient and accurate capture/retrieval/use of patient data; (2) *Redesigning EMR-supported work practices for optimal fit *- by reorganizing the clinical workflow to make full use of the advanced EMR features such as patient recalls and electronic referrals; (3) *Demonstrating value for money to encourage EMR adoption/use *- by leveraging incentives such as pay-for-performance in chronic disease management and improving patient safety thru such alerts as drug level monitoring; (4) *Having realistic expectations on the EMR implementation effort *- by setting tangible goals and putting in the time, resource and commitment needed to achieve them; (5) *Engaging patients in the process to improve the overall encounter experience - *by involving patients in using the EMR as a communication, information and decision tool before, during and after their office visit.

### Study limitations

There are limitations with this review. First, only online English articles in scientific journals were included; we could have missed studies in other languages, grey literature or hardcopies only. Second, the initial literature search was done by only one team member, which could have introduced bias in the initial screening step. Third, our conceptual model and vote-counting methods used to describe and correlate EMR use, measures and impacts were simplistic, which might not have captured the complexities involved with EMR adoption and their evaluation. In particular the vote counts of articles did not take into account the type of the study design or sample size. Fourth, caution is needed in generalizing our review findings due to the small number of studies selected. Last, our review covered a wide range of complex EMR issues, which might not have been adequately explored and fully explained.

## Conclusions

Currently there is limited positive EMR impact in physician office practice. The CA Framework, impacts, factors, findings and lessons described in this review provide the necessary components for us to make sense of EMR success in the office practice setting. This review contributes to the overall evidence base on EMR adoption/use to guide future effort.

## Methods

### Selection of studies

We had three practising physicians (MP, JB, CP) on our review team to assist in study selection and synthesis to ensure the relevance of our findings. One researcher (HB) searched two online databases - Ovid MEDLINE^® ^and CINAHL^® ^in early 2010 using search strategies prepared with the assistance of a medical librarian (RW). The search covered combinations of concepts for electronic medical record, office practice, physician and impact. We limited our search to English articles published in the last decade as they were more likely to be relevant than those from earlier periods (from 2000 to 2009). Studies in English were included if they: evaluated use of an EMR in an office-based setting; were based on original data; had physicians as primary end-users; focused on clinical functions; and reported impact on practice performance, patient outcomes, or physician-patient interactions. Studies were excluded if their EMRs were part of the hospital information systems or were a hospital ambulatory clinic settings, or if they were only survey studies. These criteria excluded Health Maintenance Organizations with integrated electronic patient records, such as the United States Veterans Health Administration. After removing duplicates from the combined MEDLINE^® ^and CINAHL^® ^searches one reviewer did the preliminary screening of all citations. Full-text review of the articles was done by two teams of two reviewers (one researcher and one physician per team). The third physician (MP) was the tie-breaker. The final article selection for analysis was done by consensus (FL, HB, MP). Corresponding authors of original articles were contacted by HB to verify the setting if needed (See Additional file 4: Detailed Search Protocol online supplement).

### Data extraction/synthesis

We grouped the selected articles into controlled and descriptive studies according to their design. Controlled studies were further grouped by type of design as experimental or observational. For each study HB extracted data on the author, country, year, study design, participants, intervention, measures and results, which were then confirmed by FL. We organized the studies into six topic areas: prescribing support, disease management, clinical documentation, work practice, preventive care, and patient-physician interaction. These topic areas have been used in the meta-synthesis by Lau et al. [15]. A study could have multiple measures and each measure could independently have a positive, neutral or negative impact. A positive impact is when an EMR was associated with an improvement in the measured effect; a neutral impact is when there was no difference; a negative impact is when the EMR had a worse effect.

To aggregate EMR impacts across studies, we used the "vote-counting" method applied in other HIS reviews [15,21,22] to tally the number of positive studies and impacts present. For controlled studies, a positive impact occurred when the measure showed a significant statistical difference between groups. For descriptive studies, we relied on the authors to report if the measure being examined had a positive impact. In studies with multiple measures, Garg's method [21] was adopted where ≥ 50% of the impacts should be positive for a study to be counted as positive. The impacts were categorized according to the dimensions of the CA Framework. They were tabulated separately for controlled and descriptive studies and then combined. Odds ratios were used to test for differences in the odds of positive impacts reported between controlled and descriptive studies under each topic before they were combined. The studies were also compared by country (based on adoption rates > 90% for Europe and Asia Pacific, and < 50% for North America reported by Schoen et al. [Exhibit-1 in ref [3]]), study design (experimental/observational/descriptive) and time period (2000-04 vs. 2005-09). Related impacts and factors were grouped and reported as key lessons that could influence EMR success. Two reviewers (FL, HB) worked independently on the aggregate analysis and reconciled the outputs through consensus afterwards. One physician (MP) on the review team provided critique to refine the synthesized findings.

## Endnotes

In this review, *physicians *include family physicians, general practitioners and specialists. *Office settings *include private offices where physicians work in solo/group practice, and interdisciplinary primary/specialty care clinics

## Competing interests

The authors declare that they have no competing interests.

## Authors' contributions

FL was main author for the manuscript and took part in the article screening and synthesis. MP assisted in writing the manuscript and served as tie-breaker in the review process. JB and CP both served as reviewers of the articles. HB conducted the article search, screening, selection and data extraction. RR assisted in the search protocol creation/revisions. All authors read and approved the final manuscript.

## Pre-publication history

The pre-publication history for this paper can be accessed here:

http://www.biomedcentral.com/1472-6947/12/10/prepub

## Supplementary Material

Additional file 1 **Appendix A**. Summary of Original Studies by Topic. Click here for file

Additional file 2 **Appendix B**. Summary of Impacts and Factors. Click here for file

Additional file 3 **Appendix C**. Comparison of Studies by Country, Design and Period. Click here for file

Additional file 4 **Detailed search protocol as online supplement**. Click here for file
