# Supplementary material for: Impact of electronic medical record on physician practice in office settings: a systematic review
Source: BMC Med Inform Decis Mak. 2012 Feb 24;12:10. doi: 10.1186/1472-6947-12-10 (PMC3315440; doi:10.1186/1472-6947-12-10)
Supplement: Additional file 1 — Appendix A. Summary of Original Studies by Topic. [file 1472-6947-12-10-S1.PDF]

Appendix A - Summary of Original Studies by Topic

Author	Country	Year	Study Design		Total Participants	Intervention(s)
**Prescribing**						
de Jong [32]	Netherlands	2009	Controlled Observational	Cross-sectional Secondary Analysis	133 PCPs* 749,811 contacts	CDSS‡ for prescribing integrated with the EMR♦
Filippi [34]	Italy	2003	Controlled Experimental	Cluster Randomized Controlled Trial	300 PCPs 15,343 patients	EMR reminder for antiplatelet drug prescribing in high-risk diabetic patients
Hollingworth [38]	USA	2007	Controlled Observational	Cross-sectional Time-motion	27 prescribers 42 staff	Prescribing via desktops or wireless laptops vs. paper
Linder [41]	USA	2007	Controlled Observational	Cross-sectional	2,528 PCPs 50,574 patients	EMR use
Martens [44]	Netherlands	2007	Controlled Experimental	Cluster Randomized Controlled Trial	53 PCPs 23 practices	CDSS with prescribing reminders integrated in to EMR (antibiotics, asthma, COPD or cholesterol lowering drugs)
Newby [50]	Australia	2003	Controlled Observational	Cohort Prospective Audit	35 pharmacies 1,667 prescriptions	Computer generated prescriptions
Schade [56]	UK	2006	Descriptive	Qualitative Interviews & Observations	PCPs Office staff	EMR use
Tamblyn [58]	Canada	2003	Controlled Experimental	Cluster Randomized Controlled Trial	107 PCPs 12,560 patients	MOXXI CDSS with alerts to identify 159 clinically relevant prescribing problems
Tamblyn [57]	Canada	2008	Controlled Experimental	Cluster Randomized Controlled Trial	28 PCPs 3,449 patients	MOXXI CDSS medication alert customization (on-demand vs. computer-triggered)
**Disease Management**						
Bassa [24]	Spain	2005	Controlled Experimental	Quasi-experimental Before-and-after No Control Group	500 patients	CDSS implementation of a practice guideline for the management of patients with hypercholesterolemia
Crosson [31]	USA	2007	Controlled Observational	Observational Cross-sectional	927 patients	EMR use
Kinn [40]	USA	2001	Controlled Experimental	Quasi-experimental Before-and-after Historical Control	26 PCPs 1,873 patients	EMR with the integrated cholesterol management tool Virtual Lipid Clinic
Mitchell [47]	UK	2004	Controlled Experimental	Cluster Randomized Controlled Trial	30,345 patients	"Rule-of-halves" practice feedback and patient-specific feedback on absolute risk of death from stroke in 10-years
Montgomery [49]	UK	2000	Controlled Experimental	Cluster Randomized Controlled Trial	27 practices 614 patients	CDSS and cardiovascular risk chart or risk chart only
Rollman [54]	USA	2001	Controlled Experimental	Cluster Randomized Controlled Trial	16 PCPs 212 patients	Interactive diagnosis e-mail alert generated throug an EMR system
van Wyk [61]	Netherlands	2008	Controlled Experimental	Cluster Randomized Controlled Trial	77 PCPs 87,886 patients	Alerting vs. on-demand CDSS intergrated with ELIAS EMR
**Clinical Documentation**						
Christensen [29]	Norway	2008	Descriptive	Qualitative Questionnaire Survey, Observations & Focus Groups	23 PCPs (focus groups) 11 PCPs (observations) 229 PCPs (survey) 24 (patient interviews)	EMR use

Hamilton [36]	UK	2003	Controlled Observational	Observational Cohort	1,396 patients	EMRs or hybrid systems vs. paper records
Hippisley-Cox [37]	UK	2003	Controlled Observational	Observational Cross-sectional	53 PCPs	Paperless medical records
Mitchell [48]	UK	2003	Controlled Experimental	Quasi-experimental Before-and-after Concurrent Control	1,882 patients	Computerized recording of chronic disease management items
Vainiomaki [59]	Finland	2007	Controlled Observational	Observational Cross-sectional	50 PCPs<br>175 consultations	EMR use
Wells [63]	New Zealand	2007	Controlled Experimental	Quasi-experimental Before-and-after No Control Group	80 PCPs<br>3,5645 patients	CDSS (PREDICT-CVD) integrated with the electronic medical record
**Work Practice**						
Bolger-Harris [25]	Australia	2008	Descriptive	Qualitative Interviews & Focus Group	31 PCPs<br>15 Other providers	General practice management plans and team care plans
Cauldwell [27]	UK	2009	Controlled Experimental	Quasi-experimental Before-and-after Concurrent Control	9 PCPs<br>100 patients<br>11 staff	Patient self-registration and medical record viewer
Christensen [28]	Norway	2008	Descriptive	Qualitative Questionnaire Survey, Observations & Focus Groups	23 PCPs (focus groups)<br>11 PCPs (observations)<br>229 PCPs (survey)<br>24 patients (interviews)	EMR use
Crosson [30]	USA	2005	Descriptive	Case Study/Series Interviews & Observations	8 providers<br>19 staff	EMR use
Dennison [33]	UK	2006	Controlled Observational	Observational Cohort	22 PCPs<br>243 patients	Electronic referral system
Keshavjee [13]	Canada	2001	Controlled Experimental	Quasi-experimental Before-and-after No Control Group	32 PCPs	EMR use
Miller [46]	USA	2004	Descriptive	Case Study/Series Interviews	20 PCPs	EMR use
Miller [45]	USA	2005	Descriptive	Case Study/Series	PCPs<br>Office staff	EMR use
Poley [51]	Netherlands	2007	Controlled Experimental	Quasi-experimental Before-and-after Concurrent Control	184 PCPs	CDSS for blood test ordering integrated into the EMR
Randeree [52]	USA	2007	Descriptive	Case Study/Series Interviews	PCPs<br>Office staff	EMR use
Robinson [53]	Australia	2003	Descriptive	Qualitative Interviews	8 PCPs	EMR use
Samoutis [55]	Greece	2007	Descriptive	Qualitative Multi-method	5 PCPs<br>5 nurses<br>18 patients	EMR use
van Wijk [60]	Netherlands	2001	Controlled Experimental	Cluster Randomized Controlled Trial	60 PCPs	CDSS for blood test ordering using BloodLink-Restricted or BloodLink-Guideline

Wager [62]	USA	2000	Descriptive	Qualitative Interviews & Observations	12 PCPs<br>54 staff	EMR use
**Preventive Care**						
Baron [23]	USA	2007	Descriptive	Case Study/Series	4 PCPs<br>1,582 patients	EMR use
Frank [35]	Australia	2004	Controlled Experimental	Randomized Individual	10 PCPs<br>10,507 patients	Electronic reminders about 12 preventive care activities
Kenealy [39]	New Zealand	2005	Controlled Experimental	Cluster Randomized Controlled Trial	107 PCPs<br>5,628 patients	Patient reminders, computer reminders or a combination of both types of reminders
**Patient-Physician Interaction**						
Booth [26]	UK	2004	Descriptive	Qualitative Videotape Analysis	10 PCPs	EMR use
Chan [12]	UK	2008	Descriptive	Qualitative Videotape Analysis & Questionnaire	10 PCPs	EMR use
Ludwick [42]	Canada	2008	Descriptive	Case Study/Series Interviews	9 PCPs	EMR use
Margalit [43]	Israel	2006	Descriptive	Qualitative Videotape Analysis	3 PCPs<br>30 patients	EMR use

* Primary Care Physician ‡ Clinical Decision Support System ♦ Electronic Medical Record
