# Supplementary material for: Impact of electronic medical record on physician practice in office settings: a systematic review
Source: BMC Med Inform Decis Mak. 2012 Feb 24;12:10. doi: 10.1186/1472-6947-12-10 (PMC3315440; doi:10.1186/1472-6947-12-10)
Supplement: Additional file 2 — Appendix B. Summary of Impacts and Factors. [file 1472-6947-12-10-S2.PDF]

Appendix B - Summary of Impacts and Factors

Source	Measures	Results	CA Framework Micro-level Dimension	Impact by Measure	Impact by Study	Factors That Influenced EMR Success
**Prescribing**						
de Jong 2009 [32] Netherlands (controlled)	Proportion of prescriptions in accordance with guidelines	CDSS users 0.89 vs. non-users 0.75, P = 0.04	NB Care Quality	Positive	Positive	**Factor1**: compliance with guidelines improves when CDS is used
	Variation in prescribing as measured by Herfindahl-Hirschman Index (HHI)	HHI CDSS users 40.3 vs. non-users 41.4, P = 0.3	NB Care Quality	Neutral		**Factor2**: CDS does not reduce variation in prescribing
Filippi 2003 [34] Italy (controlled)	Number of treated patients	Intervention vs. contol group OR 1.99	NB Care Quality	Positive	Positive	**Factor1**: physicians already using computers more likely to accept CDS
						**Factor2**: CDS must be integrated into clinical workflow
Hollingworth 2007 [38] USA (controlled)	Time efficiency of e-prescribing vs. hand written prescriptions	Additional time spent for an e-prescription = 12 seconds (not statistically significant)	NB Productivity	Neutral	Neutral	**Factor1**: workflow not disrupted, more computer but less writing tasks
						**Factor2**: no increase in combined computer/writing time for prescribers
						**Factor3**: 3-5 minutes more justified if improved safety and quality of care
Linder 2007 [41] USA (controlled)	Medical management of common diseases (eight indicators)	Six indicators showed no significant difference between groups. One indicator favoured the EMR group and one indicator favoured non-EMR group	NB Care Quality	Neutral	Neutral	**Factor1**: EHR may lack CPOE, notes, prescribing, CDS features
	Antibiotic use for acute otitis media	EMR group 68% vs. non-EMR group 67%, p = .92	NB Care Quality	Neutral		**Factor2**: physicians may not be using EMR or quality improvement focus
	Preventive counseling on smoking, diet (high-risk adults) and exercise (high-risk adults)	No significant differences	NB Care Quality	Neutral		
	Screening tests: BP check, no routine ECG, urinalysis, or Hgh/Hct	Significant difference for no routine urinalysis only, favours EMR	NB Care Quality	Neutral		
Martens 2007 [44] Netherlands (controlled)	Summary score for recommendations not to use certain drugs	No significant differences between groups: Antibiotics 28.2 vs.39.7 Asthma/COPD 1.1 vs. 2.2 Cholesterol 0 vs. 0.1	NB Care Quality	Neutral	Neutral	**Factor1**: difficulty translating guidelines into reminders
	Summary score for recommendations advocating certain drugs	No significant differences between groups: Antibiotics 20.7 vs. 20.5 Asthma/COPD 5.9 vs. 7.7 Cholesterol 1.0 vs. 1.2	NB Care Quality	Neutral		**Factor2**: technical problems and poor usability affected usage
Newby 2003 [50] Australia (controlled)	Frequency of repeat prescription ordering	Computer 69% vs. hand-written 40%, OR 3.3	NB Care Quality	Negative	Negative	**Factor1**: default settings led to increased use of antibiotics
	The proportion of people who filled their repeat prescription	Computer 61% vs. hand-written 69%, OR 0.69	NB Care Quality	Neutral		
Schade 2006 [56] UK (descriptive)	e-Prescribing, efficiency, quality of care, medicolegal	Improved documentation	IS Quality Information	Positive	Positive	**Factor1**: shift to pay for performance, subsidy to buy EMRs
		Quality care consistent, reduced error	NB Care Quality	Positive		**Factor2**: improved e-prescribing, efficiency
		e-Prescribing saves time not medical errors	NB Productivity	Positive		**Factor3**: improved documentation, standardized data
						**Factor4**: medicolegal with improved documentation
Tamblyn 2003 [58] Canada (controlled)	Potenially inappropriate prescriptions per 1000 visits	CDSS users 43.8 vs. contol 52.2, RR 0.82	NB Care Quality	Positive	Positive	**Factor1**: complete drug profile reduces inappropriate initial prescribing
	Discontinuation rate of potenially inappropriate prescriptions	Overall, no statistically significant difference between groups.	NB Care Quality	Neutral		**Factor2**: relevant clinical alerts reduce inappropriate initial prescribing
						**Factor3**: lack of treatment indications, weight, height, renal function limit effect

						**Factor4**: unclear responsibility for patient treatment in discontinuing drugs
						**Factor5**: poor technical performance affects use of CDS
Tamblyn 2008 [57] Canada (controlled)	Prevalence of prescribing problems	No significant difference in the overall prevalence of prescribing problems.	NB Care Quality	Neutral	Neutral	**Factor1**: control over alerts impacted number of alerts viewed and ignored
						**Factor2**: ignored due to known problem, benefit vs. risk, clinically irrelevant
						**Factor3**: lack medical history, age, renal, comorbidity, evidence drug risk/benefit
**Disease Management**						
Bassa 2005 [24] Spain (controlled)	Proportion of patients meetings the treatment goals	Increased 11.9%	NB Care Quality	Positive	Positive	**Factor1**: participation in algorithm development led to high adherence
	Median LDL values	Decreased 10 mg/dL	NB Productivity	Positive		
	Proportion of patients treated with drugs	Decreased 14.6%	NB Productivity	Positive		
	Mean total costs per patient	Decreased €78.4	NB Productivity	Positive		
Crosson 2007 [31] USA (controlled)	Adherence to guidelines for processes of care	OR 2.25, P < 0.001 favours Non-EMR practices	NB Care Quality	Negative	Negative	**Factor1**: attention to workflow redesign to avoid unintended consequences
	Adherence to guidelines for treatment	OR 1.67, P = 0.02 favours Non-EMR practices	NB Care Quality	Negative		**Factor2**: vendors include better features to support quality, e.g., CDM registry
	Achievement of intermediate outcomes	OR 2.68, P = 0.001 favours Non-EMR practices	NB Care Quality	Negative		
Kinn 2001 [40] USA (controlled)	Percentage of patients with documentation of low-density lipoprotein (LDL) on the chart	94% vs. 47%, P < 0.0001	IS Quality Information	Positive	Positive	**Factor1**: prompts when patient-physician communication is optimal
	Percentage of patients with LDL at or below goal (LDL < 100 mg/dl)	65% vs. 22%, P < 0.0001	NB Care Quality	Positive		**Factor2**: positive reinforcement of behavior by comparison with peers
	Percentage of patients using lipid lowering drugs	73% vs 56% p<0.001	NB Care Quality	Positive		
Mitchell 2004 [47] UK (controlled)	Proportion of hypertensives with BP recorded	Levels of recording increased in each group over the study period, but largest increase in audit-only group	IS Quality Information	Positive	Positive	**Factor1**: strategic, population patient-specific feedback help identify high-risk patients
	Proportion of controlled hypertensives	Largest proportion of controlled patients were found in the audit plus strategic feedback group vs. audit only and the control group, p = 0.028	NB Care Quality	Positive		
	Mean systolic BP	Lowest mean unadjusted systolic BP was found in the audit plus strategic feedback group	NB Care Quality	Positive		
Montgomery 2000 [49] UK (controlled)	Percentage of patients with 5 year cardiovascular risk ≥10%	CDSS group was more likely to have a cardiovascular risk ≥10% vs. chart only patients, OR 2.3	NB Care Quality	Neutral	Neutral	**Factor1**: risk chart depends on visual cue, not supported by initial CDSS
	Systolic blood pressure	The chart only group had significantly lower systolic BP vs. usual care group, mean difference -4.6 mm Hg	NB Care Quality	Neutral		
	Diastolic blood pressure	Reduction of diastolic BP did not differ between groups	NB Care Quality	Neutral		
	Prescribing of cardiovascular drugs	The chart only group were twice as likely to be prescribed two classes of cardiovascular drugs and over 3 times as likely to be prescribed three or more classes of drugs compared with the other groups	NB Care Quality	Positive		
Rollman 2001 [54] USA	Time for PCP response to the electronic message	Time to response was 1.6 times faster for PCPs in the active care vs. passive care arm	NB Productivity	Neutral	Neutral	No factors identified

(controlled)	Rate of PCP agreement with the diagnosis	No statistically significant differences across study arms	NB Care Quality	Neutral		
	PCPs' initial treatment recommendations after EMR feedback	No statistically significant differences across study arms	NB Care Quality	Neutral		
van Wyk 2008 [61]<br>Netherlands<br>(controlled)	Percentage of patients screened	CDSS alerting 65% vs. control 25%, RR 1.76<br>CDSS on-demand 35% vs. control 25%, RR 1.28<br>CDSS alerting vs. on-demand, RR 1.40	NB Care Quality	Positive	Positive	**Factor1**: display recommendations that fit workflow increases performance
	Percentage of patients treated	CDSS alerting 66% vs. contol 36%, RR 1.40<br>CDSS on-demand 40% vs. control 36%, RR 1.19<br>CDSS alerting vs. on-demand, RR 1.18	NB Care Quality	Positive		**Factor2**: alert better than on-demand, creating awareness of preventive actions
**Clinical Documentation**						
Christensen 2008 [29]<br>Norway<br>(descriptive)	Availability of information in EMR	Unsatisfied with availability of information	IS Quality System	Negative	Negative	**Factor1**: want better organization by condition
		Want dynamic e-communication	IS Quality System	Negative		**Factor2**: want e-communication with peers/patients
		Want better organization by condition	IS Quality Information	Negative		
Hamilton 2003 [36]<br>UK<br>(controlled)	Number of consultations recorded	Median in 2 years:<br>hybrid 11, EMR 9, paper 8, P < 0.001 (paper 16% lower than hybrid, EMR 11% lower than hybrid)	IS Quality System	Negative	Negative	**Factor1**: EMR include phone, missed home visits, with minimalist recording
	Number of symptom codes recorded	Paper or hybrid systems outperformed EMRs in mean number of symptom codes recorded, negative symptom recording, recording of symptom duration, and recording of symptoms potentially associated with cancer	IS Quality System	Negative		**Factor2**: need items for severity and duration of consultation
Hippisley-Cox 2003 [37]<br>UK	Absolute numbers of words, abbreviations, values, numbers and symbols recorded	Paperless records contained significantly more words, abbreviations, and symbols	IS Quality Information	Neutral	Neutral	**Factor1**: paperless record favorable but not enough significant items
(controlled)	Scoring for quality of information recorded (22 items)	Six out of 22 items had a significant difference in favour of paperless records.	IS Quality Information	Neutral		**Factor2**: no difference in recall patients/consults (both low)
	Degree of physician recall of consultations (16 items)	One out of 16 items showed a significant difference in favour of paperless records.	IS Quality Information	Neutral		**Factor3**: paperless record more words, abbreviations, legible, understandable
Mitchell 2003 [48]<br>UK<br>(controlled)	Diabetic items: BP, fundoscopy, HbA1c, height, smoking, weight, foot pulses, foot sensation	Statistically significant increase in recording of height, weight, foot pulses, and foot sensation in computer group	IS Quality Information	Neutral	Neutral	**Factor1**: increased easily recordable items during consultation
	Rheumatoid items: BP, ceratinine, CRP, ESR, FBC, RFS, urinalysis	Statistically significant increase in recording of BP and urinalysis only in computer group	IS Quality Information	Neutral		**Factor2**: effect more evident in practices with more patients
Vainiomaki 2007 [59]<br>Finland<br>(controlled)	Quality of patient records relative to legislation	18% poor, 62% moderate, 20% good	IS Quality Information	Negative	Negative	**Factor1**: quality of patient record depends on EMR used, not consultation quality
	Quality of patient records relative to experienced quality of consultation	No correspondence	IS Quality Information	Neutral		**Factor2**: legislation requirements too high for PCPs with heavy workload
	Quality of patient records relative to the electronic system	Quality of patient records found to change according to the electronic system employed	IS Quality Information	Negative		**Factor3**: difficulty in recording encounter when patients have many problems
Wells 2007 [63]<br>New Zealand<br>(controlled)	Proportion of patients with a documented CVD risk	Before 2.8% after 10.7%,<br>RR 4.0	IS Quality Information	Positive	Positive	**Factor1**: personalized IT training/support at no charge, by GPs
	Completeness of CVD risk factor documentation	Before 9.2% after 18.0%,<br>RR 1.9	IS Quality Information	Positive		**Factor2**: payment per risk assessment offset setup/ongoing costs
**Work Practice**						

Bolger-Harris 2008 [25]<br>Australia<br>(descriptive)	GP users-freq, disease, use/improv<br>GP nonusers - reasons, aware, adopt?<br>providers-freq, format, align, care coord	Providers-diff TCA/coordination	NB Productivity	Postitive	Positive	**Factor1**: templates alone not improve care coordination
		GPs used templates for GPMP, TCA	NB Productivity	Positive		**Factor2**: GPs can benefit from templates for billing
Cauldwell 2009 [27]<br>UK<br>(controlled)	Patient registration time	Users: 1/15 > 1 minute<br>Non-users: mean 1 min. 53 sec.<br>Control: 3/47 > 1 minute	NB Productivity	Positive	Positive	**Factor1**: need to address patient acceptance of technology
	Average clinical consultation time	Users: 10.11 minutes<br>Non-users: 12.4 minutes<br>Control: 11.5 minutes	NB Productivity	Neutral		**Factor2**: patient self-register but staff had to show patients how to use system
Christensen 2008 [28]<br>Norway<br>(descriptive)	EPR function, time, GP-patient relation	EMR available improved not content	IS Quality Information	Neutral	Neutral	**Factor1**: redundant content, lack overview
		EMR use efficient/comprehensive	NB Productivity	Positive		**Factor2**: improved access to record
		Admin work from staff to GP	US Use/Satisfaction	Neutral		**Factor3**: reuse info but shift in admin tasks to GPs
		No GP-patient relation disturbance	US Use/Satisfaction	Neutral		**Factor4**: EMR used before, after and during encounter no disturbance
Crosson 2005 [30]<br>USA<br>(descriptive)	Communication, EMR use<br>DS authority, resolving conflicts	Dysfunctional communication	NB Productivity	Negative	Negative	**Factor1**: culture affect EMR use to improve care
		Authority distribution, conflicts	NB Productivity	Negative		**Factor2**: commmunication, decision making, conflict
		Preventive health, CDM unwieldy	NB Productivity	Negative		
Dennison 2006 [33]<br>UK<br>(controlled)	Waiting time from GP referral until appointment booked	Median days electronic 0 vs. paper 7	NB Productivity	Positive	Positive	**Factor1**: patient has choice of dates, greater responsibility to attend
	Waiting time from GP referral to actual clinic appointment	Median days electronic 8 vs. paper 10	NB Productivity	Neutral		
	Patient attendance rate in clinic	Non-attendance rate electronic 8.5% vs. paper 22.5%	NB Productivity	Positive		
Keshavjee 2001 [13]<br>Canada<br>(controlled)	Time to perform six administrative tasks	Time required to perform 5/6 administrative tasks decreased at 6 months and 4/6 at 18 months post-implementation	NB Productivity	Neutral	Neutral	**Factor1**: increased charting time nurses given more responsibility
	Time to perform six PCP tasks	Time required to perform 4/6 PCP tasks had not decreased at 6 months, but 3/6 had decreased by 18 months post-implementation.	NB Productivity	Neutral		**Factor2:** physician chart time decreased but may be due to drop-outs
						**Factor3**: implement success from managing stress, perceiving value
						**Factor4**: cognitive dissonance - more charting, more patients, leaving earlier
Miller 2004 [46]<br>USA<br>(descriptive)	Functions installed, used, organizational change, time, costs, finanacial/quality benefits	Less time, staffing, transcription	IS Quality System	Positive	Positive	**Factor1**: cost savings
		Guideline care thru templates	IS Quality Information	Positive		**Factor2**: improved quality of care with guidelines
		More documentation/code<br>Use template	NB Care Quality<br>NB Productivity	Positive<br>Positive		
Miller 2005 [45]<br>USA<br>(descriptive)	EMR use, financial costs/benefits, QI payback, risk, time cost/benefits, QoL	Improved billing/access, less staff/transcription	IS Quality Information	Positive	Positive	**Factor1**: led to cost savings/profits
		Improved documentation	NB Care Quality	Positive		**Factor2**: improved documentation
			NB Productivity	Positive		**Factor5**: improved QI, reminders
						**Factor3**: need more pay-for-performance

						**Factor4**: depends on champion, process change, culture
Poley 2007 [51] Netherlands (controlled)	Number of order forms	No statistically significant reduction in the number of order forms	NB Productivity	Positive	Positive	**Factor1**: use affected by financial incentives, labs get less reimbursement
	Number of tests per order form	Tests ordered per form decreased in the intervention group by 6%, P = 0.001	NB Productivity	Positive		**Factor2**: address practical logistics with order forms with different solutions
	Costs of laboratory requests	Mean cost decrease of 3% in the intervention group vs. 2% increase in the control group, P = 0.009	NB Productivity	Positive		**Factor3**: check for substitution effect, e.g., more medications, PCP/specialist consults
Randeree 2007 [52] USA (descriptive)	Actual vs. expected costs and benefits items that increase budget themes before/after adoption	Vendor, contract train, time, productive	IS Quality System	Negative	Negative	**Factor1**: implementation - planning, downtime, workflow, maintenance cost
		Vendor, contract, staff, oversite, buy-in	IS Quality Service	Negative		
		Vendor, rework, staff, training, time	NB Productivity	Negative		
		Budget, workflow, expectation				
Robinson 2003 [53] Australia (descriptive)	IT use -EMR, web, email, store-forward, videoconferencing, e-discussion groups, value of EMR	Most EMR/web, increase e-mail/SF	NB Productivity	Positive	Positive	**Factor1**: need for training, tech support
		No videoconferencing, e-discussion groups	NB Productivity	Neutral		**Factor2**: lack time to learn EMR
						**Factor3**: videoconf/discussion gps little value, too complicated
Samoutis 2007 [55] Greece (descriptive)	User satisfaction, barriers, actions, use, expectation, support, impact	Lab, prescribe, reminders, coding	IS Quality System	Positive	Negative	**Factor1**: lacked pay-for-performance incentives
		Poor usability	IS Quality System	Negative		**Factor2**: need workflow integration with hospital
		Increased workload	NB Productivity	Negative		**Factor3**: need paper for legal concerns
		Patient not negative toward EMR	US Use/Satisfaction	Neutral		**Factor4**: transition difficulties, lack familiarity with
						**Factor5**: system breakdown software design problems
van Wijk 2001 [60] Netherlands (controlled)	Average number of tests per order form per practice	PCPs who used Bloodlink-Guideline ordered 20% fewer tests per form than PCPs who used Bloodlink-Restricted, P = 0.003	NB Productivity	Positive	Positive	**Factor1**: guideline does not reduce all test ordering to same degree
Wager 2000 [62] USA (descriptive)	Perceived benefits/limits of EMR Organization impact of EMR on practice Implementation process -CSF	Better doc, organized, legible, complete	IS Quality Information	Positive	Positive	**Factor1**: leader, training, resource, commitment
		Access, efficiency, communication	NB Productivity	Positive		**Factor2**: Downtime, backup, technical support
		Improved manament patient records, care, jobs	NB Productivity	Positive		**Factor3**: extent of hybrid paper-EMR system
		Improved communication, work environment	NB Productivity	Positive		
**Preventive Care**						
Baron 2007 [23] USA (descriptive)	Mammography screening rates	10% absolute improvement in rate	NB Care Quality	Positive	Positive	**Factor1**: need for structured data, standards,
						**Factor2**: restructuring primary teams more resources
Frank 2004 [35] Australia (controlled)	Uptake of preventive care opportunities	Relative changes in four of the 12 preventive activities favoured the use of electronic reminders	NB Care Quality	Neutral	Neutral	**Factor1**: fee-for-service payment with no rebates for preventive care
						**Factor2**: possible information overload from multiple reminders
						**Factor3**: reminders made PCPs aware of preventive care for all their patients

						**Factor4**: reminders displayed by date when due rather than priority
Kenealy 2005 [39]<br>New Zealand<br>(controlled)	Eligible patients screened for diabetes	Computer vs. usual care OR 2.55<br>Patient reminders vs. usual care OR 1.72<br>Both vs. usual care OR 1.69<br>Computer vs. patient OR 1.49	NB Care Quality	Positive	Positive	No factors identified
**Patient-Physician Interaction**						
Booth 2004 [26]<br>UK<br>(descriptive)	EMR use before, after and during consultations	Avoid multitasking, summary assess	US Use/Satisfaction	Neutral	Neutral	**Factor1**: skill/attention to manage screen/consultation
		Behavior-control, ignore,responsive	US Use/Satisfaction	Neutral		**Factor2**: rapport thru joint use computer by patient/GP feasible
		Transition-signpost, chatter, respond	US Use/Satisfaction	Neutral		**Factor3**: strategies - signposting, chatter, responding
Chan 2008 [12]<br>UK<br>(descriptive)	Time: consultation length, patient-doctor interaction, computer, patient-doctor-computer interact	End, continuous, minimal users	US Use/Satisfaction	Positive	Positive	**Factor1**: can adjust psych consult sessions
		Tailored psych consults	US Use/Satisfaction	Positive		**Factor2**: 3 styles of EMR use - end, continuous, minimal
Ludwick 2008 [42]<br>Canada<br>(descriptive)	Exp select, implement, operate EMR sociotechnical factors for implementation success	Issue-time, training, support, learn	IS Quality System	Positive	Positive	**Factor1**: struggled, benefit with more time investment
		Disrupt information flow, room/EMR layout	IS Quailty Service	Negative		**Factor2**: visual cue, room layout important for interaction
		Template use data entry	US Use/Satisfaction	Neutral		**Factor3**: templates for common data entry, efficiency
			NB Productivity	Positive		
Margalit 2006 [43]<br>Israel<br>(descriptive)	Screen gaze time, keystrokes<br>length of visit, visit dialog	Gaze reduces dialog psych/emotional	US Use/Satisfaction	Neutral	Neutral	**Factor1**: can reduce dialog in psych/emotion consultations
		Keyboarding increases biomedical data	US Use/Satisfaction	Neutral		
