# Supplementary material for: Impact of electronic medical record on physician practice in office settings: a systematic review
Source: BMC Med Inform Decis Mak. 2012 Feb 24;12:10. doi: 10.1186/1472-6947-12-10 (PMC3315440; doi:10.1186/1472-6947-12-10)
Supplement: Additional file 3 — Appendix C. Comparison of Studies by Country, Design and Period. [file 1472-6947-12-10-S3.PDF]

Appendix C - Comparison of Studies by Country, Time Period and Study Design

By Country	Positive	Neutral	Negative	Total	OR	CI
North America [1]	7	5	3	15		
Europe, Asia Pacific [2]	14	7	3	24		
Excluded [3]	1	1	2	4		
Total	22	13	8	43	1.6	0.44 - 5.87

[1] United States, Canada with adoption rates <50%

[2] United Kindgom, The Netherlands, Australia, New Zealand, Norway, Italy with adoption rates >90%

[3] Spain, Finland, Greece, Israel - these countries not listed in Schoen et al. ref 3 exhibit 1

By Time Period	Positive	Neutral	Negative	Total	OR	CI
Years 2000-2004	8	7	2	17		
Years 2005-2009	14	6	6	26		
Total	22	13	8	43	1.31	0.39 - 4.47

By Study Design	Positive	Neutral	Negative	Total	OR	CI
Controlled-Experimental	11	7	0	18		
Controlled-Observational	2	3	4	9		
**Total**	13	10	4	27	5.5	0.88 - 34.46
