# Supplementary material for: Impact of electronic medical record on physician practice in office settings: a systematic review
Source: BMC Med Inform Decis Mak. 2012 Feb 24;12:10. doi: 10.1186/1472-6947-12-10 (PMC3315440; doi:10.1186/1472-6947-12-10)
Supplement: Additional file 4 — Detailed search protocol as online supplement. [file 1472-6947-12-10-S4.PDF]

Impact of EMR on Physician Practice in Office Settings: A Systematic Review Detailed Search Protocol

Search Strategy

A scoping search for relevant studies was carried out in May 2009; results were refined with the assistance of a medical librarian to create a more comprehensive search strategy. Test searches revealed that a broad strategy was required due to variations in study indexing. Because the final search strategies returned large volumes of records, we limited the scope of our primary literature search to two online databases – Ovid MEDLINE® and CINAHL®. We also limited our search to the 2000 – 2009 publication years in order to capture a more current view of the office-based physician's environment. Both database searches were completed in June 2009. In addition, we carried out a search for unpublished membership surveys related to EMR use by Canadian physicians. We searched the Websites of all Federal and Provincial colleges of physicians and surgeons; the Canadian Medical Association (CMA) and all provincial medical association Websites; and Websites of all CMA affiliates and national specialist societies. Searches were limited to the publically accessible portions of the Websites. Details of this supplementary search can be obtained from the corresponding author.

Lastly, the references of 29 systematic reviews know to the authors and related to health information technology were hand searched for potentially relevant original studies [see reference 15]

Selection of Studies

Studies were eligible for our review if they met 5 inclusion criteria. First, the study had to evaluate the impact of implementing and/or using an EMR system on a specific outcome; second, we only included studies based on original data; third, the primary end-user of the EMR had to be a physician; fourth, the EMR system had to support clinical (not just administrative) functions; and finally, the outcome measure(s) reported had to be related to how the EMR either positively or negatively affected practitioner performance, patient outcomes, or practitioner/patient attitudes.

Moreover, studies were rejected if they met either one of two exclusion criteria. First, we excluded studies of outpatient EMRs that were integrated with inpatient information systems. This resulted in the exclusion of Health Maintenance Organizations (HMOs) with integrated electronic patient records and other large networks such as the Veterans Health Administration in the United States. Second, we excluded studies where the site was a hospital-based outpatient clinic.

After removing duplicate records from the combined search strategies, one reviewer carried out the preliminary screening of all unique citations. Full-text review of the remaining papers was carried out by two teams of two reviewers and the final selection of papers for analysis was by consensus. Corresponding authors were contacted to verify the study setting when necessary.

In keeping with the exploratory nature of our research, all types of study designs were eligible for inclusion in our review. As a result, our final selections included analytic (comparative) studies, descriptive studies, and surveys. Analysis of the surveys is in process and will be reported on separately.

Complete MEDLINE® Search Strategy with Results

Ovid MEDLINE® In-Process & Other Non-Indexed Citations and Ovid MEDLINE® 1950 to Present were accessed through the University of Victoria Libraries portal on June 15, 2009, and searched using OvidSP®. Following is a detailed summary of the final search strategy and search results:

	**Final Search Terms with Medline®/OvidSP® Commands**	**Results**
1.	exp Medical Informatics/	213,005
2.	exp Computers/ut	1,012
3.	Drug Therapy, Computer-Assisted/	1,129
4.	Electronic Prescribing/	42
5.	EMR.ti,ab.	1,798
6.	EHR.ti,ab.	902
7.	EPR.ti,ab.	11,861
8.	computer$.ti	52,363
9.	paperless.ti,ab.	297
10.	(electronic adj2 record?).ti,ab.	5,023
11.	1 OR 2 OR 3 OR 4 OR 5 … OR 10	265,042
12.	exp Ambulatory Care Facilities/	35,895
13.	exp Group Practice/	21,687
14.	exp Practice Management, Medical/	7,800
15.	exp Specialties, Medical/	398,596
16.	exp Ambulatory Care/	39,533
17.	exp Primary Health Care/	56,084
18.	Physician's Offices/	1,255
19.	Physician's Practice Patterns/	28,150
20.	Professional Practice/	12,954
21.	exp Private Practice/	8,777
22.	physicians/ or foreign medical graduates/ or occupational health physicians/ or physicians, family/ or physicians, women/	67,332
23.	Outpatients/	5,652
24.	(group adj practice?).ti,ab.	3,385
25.	(general adj practi$).ti,ab.	48,090
26.	GP?.ti,ab.	49,048
27.	(professional adj practice?).ti,ab.	2,446
28.	(office adj practice?).ti,ab.	1,019
29.	(primary adj care).ti,ab.	47,280
30.	(out adj2 hour?).ti,ab.	997
31.	(medical adj office?).ti,ab.	1,507
32.	clinic?.ti,ab.	162,021
33.	12 OR 13 OR 14 OR 15 OR 16 … OR 32	826,778
34.	11 AND 33	25,120
35.	limit 34 to yr="2000 – 2009"	14,224

Complete CINAHL® Search Strategy with Results

CINAHL® with Full-Text was accessed through the University of Victoria Libraries portal on June 13, 2009, and searched using EBSCOHost® in Boolean/Phrase mode. Following is a detailed summary of the final search strategy and search results:

	Final Search Term with CINAHL®/EBSCOHost® Commands	Results
1.	MH "Health Information Systems+"	14,592
2.	MH "Computerized Patient Record"	4,276
3.	MH "Medical Informatics"	916
4.	MH "Patient Record Systems”	2,642
5.	“EHR”	413
6.	“EMR”	407
7.	“EPR”	115
8.	TI “computer*”	8,545
9.	“paperless”	159
10.	“electronic” N1 “record*”	1,867
11.	1 OR 2 OR 3 OR 4 OR 5 … OR 10	22,923
12.	MH “Outpatients”	22,491
13.	MH “Ambulatory Care”	4,348
14.	MH “Ambulatory Care Facilities”	2,228
15.	MH “Physicians+”	32,967
16.	MH “Specialties, Medical+”	21,780
17.	MH “Practitioner’s Office”	734
18.	MH “Office Visits”	1,376
19.	MH “Private Practice”	2,302
20.	MH “Primary Health Care”	18,205
21.	“group practice”	1,069
22.	“general practi*”	7,293
23.	“GP?”	3,478
24.	“professional practice”	15,543
25.	“office practice”	130
26.	“primary” N1 “care”	30,150
27.	“out of hour?”	257
28.	“medical office?”	185
29.	“clinic?”	8,754
30.	12 OR 13 OR 14 OR 15 OR 16 … OR 29	126,806
31.	11 AND 30	2,779
32.	Filter by publication date: 2000 – 2009	2,273

Detailed Inclusion Criteria

Evaluation of EMR: We included any type of study that evaluated the impact of implementing and/or using an EMR system on a specific outcome. The EMR had to be one of the interventions in the study or one of the independent variables. If the EMR system was used only for data retrieval or data validation the study did not meet this criterion.

Primary Study: We only included studies based on original (primary) data. Studies that did not collect original data or studies that consolidate primary data from other studies (for example, systematic reviews) did not meet this criterion.

Physician End-user: The primary end-user of the EMR had to be a physician. A physician for the purpose of this review was any type of medical or surgical specialist, primary care physician or general practitioner. EMRs used mainly by other types of clinicians or office staff did not meet this criterion.

Clinical Functionality: The EMR system had to support clinical functions, for example, charting, prescribing, and decision support. Studies of EMRs used only for administrative purposes (billing or scheduling) did not meet this criterion.

Impact on Office Practice: The outcome measure(s) reported had to be related to how the EMR either positively or negatively affected practitioner performance, patient outcomes, or practitioner/patient attitudes.

Detailed Exclusion Criteria

Hospital Information System: We excluded studies of outpatient EMRs that were integrated with inpatient information systems. This resulted in the exclusion of Health Maintenance Organizations (HMOs) with integrated electronic patient records and other large networks such as the Veterans Health Administration in the United States.

Hospital Outpatient Clinic: We excluded studies where the site was a hospital-based outpatient clinic.
